# Self-Localization of Mobile Robots Using a Single Catadioptric Camera with Line Feature Extraction

**DOI:** 10.3390/s21144719

**Published:** 2021-07-09

**Authors:** Huei-Yung Lin, Yuan-Chi Chung, Ming-Liang Wang

**Affiliations:** 1 Department of Electrical Engineering, Advanced Institute of Manufacturing with High-Tech Innovation, National Chung Cheng University, Chia-Yi 621, Taiwan; 2 Department of Electrical Engineering, National Chung Cheng University, Chia-Yi 621, Taiwan; yuanchi@godel.ee.ccu.edu.tw (Y.-C.C.); minliang@godel.ee.ccu.edu.tw (M.-L.W.)

**Keywords:** catadioptric camera, omnidirectional image, robot self-localization

## Abstract

This paper presents a novel self-localization technique for mobile robots using a central catadioptric camera. A unified sphere model for the image projection is derived by the catadioptric camera calibration. The geometric property of the camera projection model is utilized to obtain the intersections of the vertical lines and ground plane in the scene. Different from the conventional stereo vision techniques, the feature points are projected onto a known planar surface, and the plane equation is used for depth computation. The 3D coordinates of the base points on the ground are calculated using the consecutive image frames. The derivation of motion trajectory is then carried out based on the computation of rotation and translation between the robot positions. We develop an algorithm for feature correspondence matching based on the invariability of the structure in the 3D space. The experimental results obtained using the real scene images have demonstrated the feasibility of the proposed method for mobile robot localization applications.

## 1. Introduction

To make the mobile robot have the ability to navigate autonomously, it is commonly equipped with various sensors for environment perception. Thus, the sensing techniques are essential to many mobile robot navigation applications, such as the self-localization, obstacle detection and avoidance, human–machine interaction, and object handling, etc. In the past few decades, a number of techniques and algorithms have been proposed to accomplish these tasks. The technological development mainly depends on the types of sensors adopted for mobile robot navigation. Currently, some widely used sensors include sonars, laser rangefinders, odometers, GPS (global positioning system), infrared sensors, and cameras. Nevertheless, GPS is usually not applicable to indoor environments and some satellite signal denied areas. This might include the application scenarios for extra-planetary rovers and under-ground robots.

The ultrasonic sonar sensor is one of the most adopted low-cost sensing devices. It is commonly used for distance sensing of the surrounding environment. However, there are some critical drawbacks such as the slow response compared to other sensors and less accurate measurements for corners and short distances. On the other hand, the laser rangefinder is probably the fastest and more accurate commercial device for depth perception. In addition to the high cost, its sensing distance range is usually limited to one-dimensional and requires extra mechanisms for multi-dimensional scans. Moreover, the laser-based technology is not able to deal with highly reflective or transparent surfaces such as glass. The odometer uses the optical encoder on the wheel to calculate the travel distance of the mobile robot. It is generally a built-in device for the wheeled robots, but the error accumulation due to the proprioceptive sensing nature makes the drifting inevitable for long range movement. Thus, the odometry is usually combined with the information from other devices, such as sonar or camera, and utilizes the sensor fusion techniques to improve the localization accuracy. There also exists an indoor self-localization technique based on the ambient magnetic field [1]. It has the advantage that the magnetic field is not sensitive to many environmental changes, which might affect the vision or range finder-based approaches. Among the current sensor technologies used for robot navigation, the cameras provide the most diverse and rich content for the environmental perception. The vision-based techniques are developed for low-level image feature extraction and then adopted for various applications such as distance measurement, robot self-localization, object recognition, scene understanding, etc.

In this work, we present a self-localization technique for mobile robots using an omnidirectional vision system. A catadioptric camera is adopted for image acquisition of the surroundings in an indoor environment. The vertical features are extracted from the image sequences, and the 3D information is derived based on the catadioptric image formation model. Different from the conventional stereo vision techniques, the feature points are projected onto a known planar surface and the plane equation is used for depth computation. The self-localization is then calculated using multiple 3D features and the imaging geometry. Furthermore, the 2D environment map and robot motion trajectory can be constructed by matching the static environment features among the image frames sequentially. Compared to other sensing techniques, such as using lidar for depth measurement, the vision-based methods adopt low-cost camera equipment for data acquisition. The acquired images can also provide rich information for other recognition and human-machine interaction tasks.

**Figure 1:**
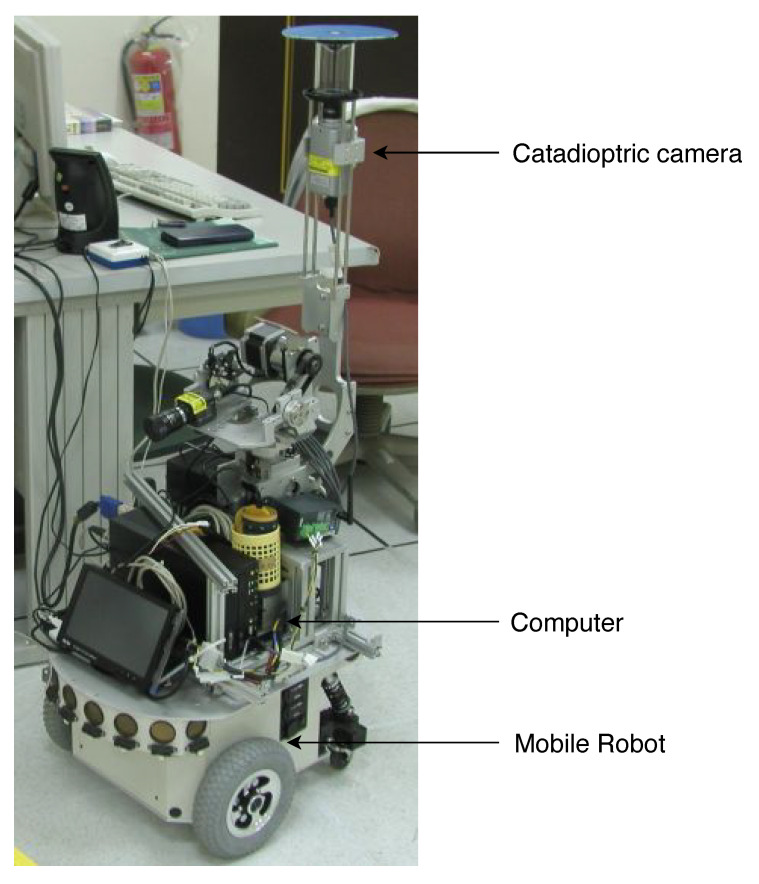
The mobile robot platform used in the experiments. The catadioptric camera mounted on the top of the robot is used for robot self-localization and pose estimation.

As illustrated in Figure 1, a catadioptric camera is adopted in this work. It consists of a hyperbolic mirror mounted on top of a conventional camera. The images captured by the omnidirectional cameras are distorted by reflection from the hyperbolic mirror and refraction from the lens. Our catadioptric camera system is calibrated using a unified sphere model for the mobile self-localization task [2]. The imaging model possesses the geometric characteristics that can be combined with the ground plane constraint for position estimation. To deal with the localization problem, we investigate the geometric derivation of the catadioptric camera model. The basic idea is to use the vertical line structures in the environment such as walls, buildings or trees to calculate the camera position. Although there is a limitation on the perpendicularity with respect to the ground plane, the vertical lines can be easily detected in the omnidirectional images. Assuming the height of the mobile robot is known, a point lies on the ground plane, and its 3D coordinates can then be estimated via the unified sphere model. Since the location estimation is based on the ground point detection, the proposed method can be extended to the robot navigation in an unknown environment with no pre-defined landmarks. The experiments carried out on the real-world scenes have demonstrated the feasibility of our self-localization approach using the catadioptric vision system and image-based ground plane detection.

## 2. Related Work

The omnidirectional vision systems have been widely adopted in the mobile robot research for simultaneous localization and mapping (SLAM) [3]. In addition to the localization accuracy, it is also an important issue to consider the applicability to a wide range of real-world scenarios. Many systems are designed for applications in pre-established environments, including the installation of artificial landmarks (or beacons) or the use of specialized sensors for some specific environments. Under these restrictions, the development of localization techniques becomes more difficult for the unknown scenes. In the early research, Grimson and Lozano-Perez utilized simple constraints between the sensor data and a preset model for object recognition and localization [4]. The constraints were then extended by Drumheller for mobile robot localization using sonar sensors [5]. Since then, many approaches have been conducted to deal with the localization problem using various sensors, such as laser rangefinders [6], odometers [7], infrared sensors [8], etc.

The self-localization of mobile robots is still an ongoing research topic due to the uncertainties of sensor data and a dynamic world. A few techniques cooperated with environment settings, such as the use of UWB (ultra-wideband) beacons and RFID tags, have been proposed in the past few decades [9,10]. In addition to the use of essential built-in sensing devices for the mobile robots, most recent works emphasize the development of image-based techniques to improve the localization accuracy while providing the environmental perception capability. The computer vision and machine learning algorithms are commonly adopted and integrated with other sensor information from IMU, odometry and GPS for robot self-localization [11]. For the approach exclusively based on the image data, it is generally considered as visual odometry or vision-based simultaneous localization and mapping (visual SLAM) [12]. The classic work presented by Nistér et al. utilized a stereo vision system to estimate the camera motion trajectory [13]. A similar concept is further extended to the direct and semi-direct methods for visual odometry using monocular and multiple camera systems [14]. In the visual SLAM research, Silveira et al. proposed a second-order approximation method to compute the localization parameters efficiently [15]. More recently, the RGB-D cameras were adopted for the development of dense and sparse SLAM techniques [16,17].

Among various vision-based localization approaches in the existing literature, the ground plane has been successfully adopted for many applications. In [18], Simond et al. used the ground plane detection and homography transformation to estimate the egomotion of a vehicle. Lobo et al. presented a technique to use stereo vision and the ground plane to assist the robot navigation [19]. Pears and Liang used corner tracking and image homographies to find coplanar corners [20]. They were then used to find the ground plane and improve the robot navigation. Renno et al. proposed a ground plane tracker (GPT) for surveillance applications [21]. A robust tracking system was constructed based on the knowledge of the ground plane detection result. In [22], Bose et al. presented an automatic technique to recover the affine and metric properties of a ground plane. They first identified the objects with constant velocities in the trajectories and then used the information to compute the transformation between the image plane and the ground plane. For other applications of ground planes, Lu et al. proposed a technique for robot navigation with the detection of curbs, stairways and obstacles [23].

Nowadays, an increasing number of mobile robots adopt a single omnidirectional camera for environment perception to take advantage of its large field of view. However, this makes the self-localization a more difficult task by using the omnidirectional images. Marques et al. designed a self-localization method using pre-calibrated image distortion parameters to warp the omnidirectional image for computation [24]. Liu et al. presented a technique to estimate the junction edges between walls and the ground plane using corner detection and preset landmarks [25]. Calabrese et al. utilized known landmarks on the ground plane and presented a triangulation-like method to solve the self-localization problem [26]. Most of the algorithms do not contain geometric analysis, i.e., they do not rely on the constraints of the environment structures to make the self-localization more accurate. This paper presents a technique to estimate the location of the mobile robot using a catadioptric camera and geometric constraint. Our approach is different from the methods based on the full 3D reconstruction of the scene [27] or the techniques implemented using sparse image matching [28]. We utilize the geometric constraints based on the feature points lying at the intersections of the ground plane and vertical lines in the environment.

## 3. Unified Central Catadioptric Model

To localize the mobile robot using only an onboard catadioptric vision system, it is required to construct the camera projection model so that the 3D scene points can be projected onto the image plane. Thus, the catadioptric camera calibration has to be carried out first to establish the geometric representation. In the last decades, several types of omnidirectional cameras have been designed, and corresponding calibration techniques have also been proposed. This work follows Mie’s presentation for the catadioptric camera model construction [29]. Since the radial distortion and decentering distortion are considered simultaneously, the proposed sphere camera model is able to provide more precise measurement results [30,31].

**Figure 2:**
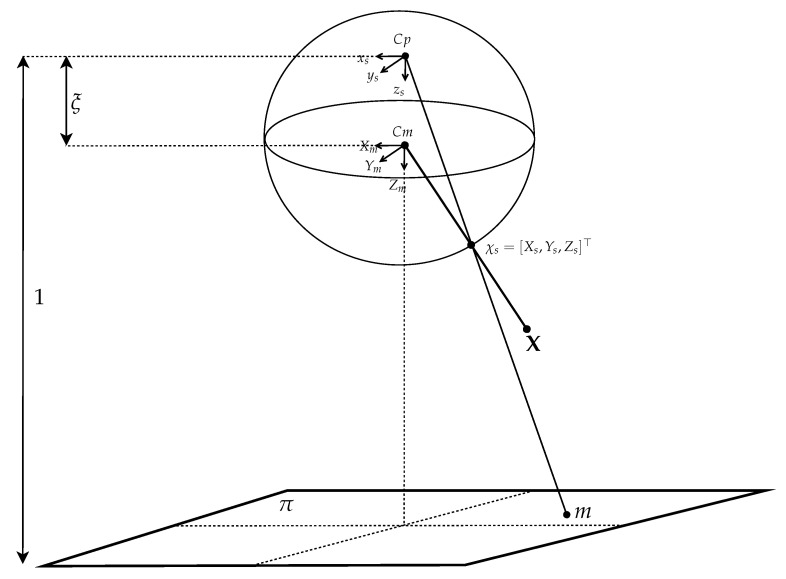
The unified sphere projection model for the catadioptric camera. Given a 3D point X, it is projected to a point on the unified sphere at $\chi_{s}$. This point is then projected to another point *m* on the image plane π via the new projection center at $C_{p}=\left(0,0,\zeta \right)$.

The unified sphere projection model for the catadioptric camera is illustrated in Figure 2. Given a 3D point X, it is projected to a point on the unified sphere at

(1) $$\chi_{s}=\frac{X}{\left||X|\right|},$$

where $\chi_{s}={\left[X_{s},Y_{s},Z_{s}\right]}^{⊤}$. This point is then projected to another point

(2) $$m=\left(X_{s},Y_{s},Z_{s}+\zeta \right)$$

on the image plane π via the new projection center (i.e., the center of the unit sphere model) at $C_{p}=\left(0,0,\zeta \right)$, where ζ is the distance between the sphere and image projection center. Thus, the point projection through the unit sphere model is given by

(3) $$p=\left[\begin{array}{c} x_{1}\\ x_{2}\\ x_{3} \end{array}\right]=Km=\left[\begin{array}{ccc} \gamma & \gamma s & u_{0}\\ 0 & \gamma r & v_{0}\\ 0 & 0 & 1 \end{array}\right]m$$

The above projection involves a generalized camera projection matrix *K* with the focal length γ, the principal point $\left(u_{0},v_{0}\right)$, the skew factor *s*, and the aspect ratio *r*. Based on the camera projection model, an image pixel $\left(x_{im},y_{im}\right)$ on an omnidirectional image is given by the ratios

(4) $$\begin{array}{cc} x_{im} & =x_{1}/x_{3}\\ y_{im} & =x_{2}/x_{3} \end{array}$$

**Figure 3:**
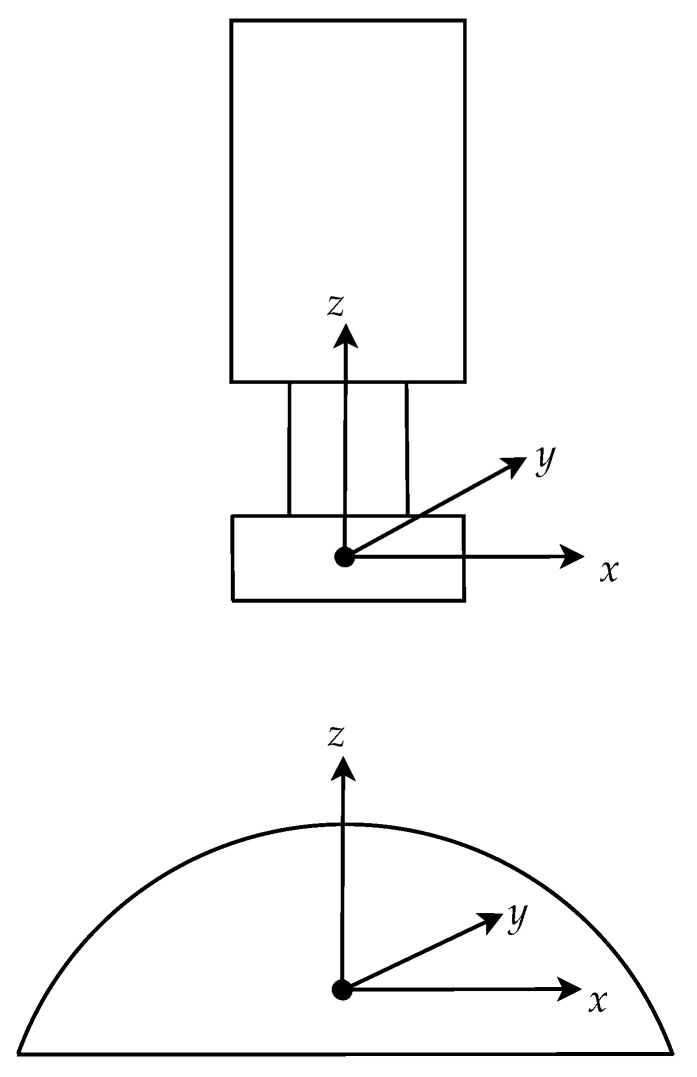
The coordinate frame of the catadioptric camera system used to represent the robot motion.

Now, suppose a feature point is given on an omnidirectional image, it can be back-projected onto the sphere using the unified projection model. Each point on the sphere can also be thought as a line radiated from the center. After the catadioptric camera is calibrated, the robot motion is represented with the camera coordinates, as shown in Figure 3.

**Figure 4:**
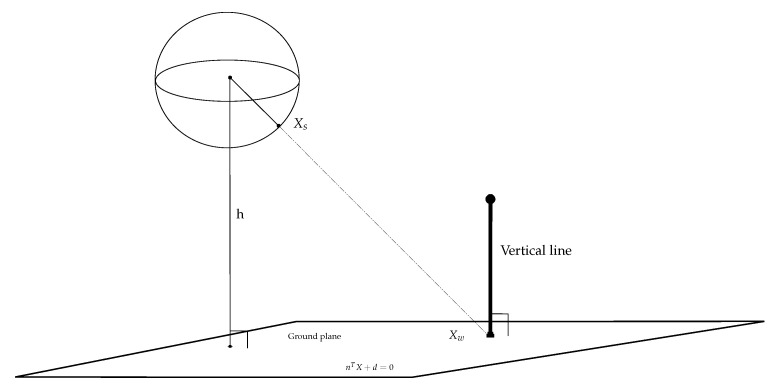
The image projection for the detection of the intersection (base point) from the corresponding vertical line.

To formulate the derivation of mobile robot self-localization, we first establish the geometric constraint of a single catadioptric camera with a sphere model and ground features. The unified sphere imaging model, the ground plane, and a vertical line representing the feature in the scene are depicted in Figure 4. The 3D point $X_{w}$ given by the intersection of the vertical line and the ground plane is projected to the surface point $X_{s}$ on the sphere model by Equations (1)–(4) with a scale factor λ. That is,

(5) $$X_{w}=\lambda {\left[X_{s},Y_{s},Z_{s}\right]}^{⊤}=\lambda \chi_{s},\;\lambda \in R$$

Since the point given by the above equation also lies on the ground, it needs to satisfy the ground plane equation

(6) $$n^{⊤}X_{w}+d=0$$

where n is the normal vector of the ground plane, and d is the distance to the camera projection center on the plane.

In the implementation, the plane equation is derived from the camera calibration. A chess-board pattern is placed on the ground, and the corner points are used to compute the normal vector n and the off-set distance d. Furthermore, since the height of the mobile robot is a constant, the scale factor λ can be calculated by

(7) $$\lambda =\frac{h}{Z_{s}}$$

where *h* is the height between the ground plane and the projection center (as shown in Figure 4). After the parameter λ is derived, $X_{w}$ is given by

(8) $$X_{w}=\lambda \chi_{s}$$

Thus, when a mobile robot identifies a number of the intersection points on the ground, they can be used to estimate the 3D information by the geometric constraints.

## 4. Ground Plane Features Detection and Robot Self-Localization

The proposed mobile robot self-localization technique consists of three steps. First, the region segmentation is carried out to extract the ground plane in the omnidirectional image. Second, the vertical lines in the scene are detected and used to identify the intersection points between lines and the ground plane. Third, the 3D coordinates of the intersection points with respect to the mobile robot are derived using the unified sphere camera model. The ground plane constraint and catadioptric projection models are used to solve the localization problem.

### 4.1. Ground Region Extraction and Vertical Line Identification

**Figure 5:**
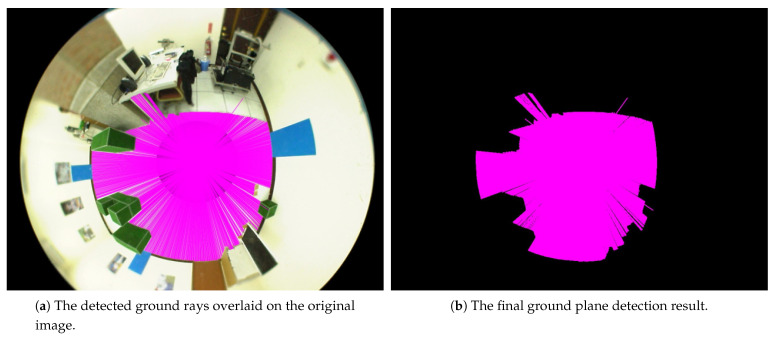
The detection of the ground plane region from the omnidirectional image using edge detection and the rays radiated from the image center.

To extract the ground region of the scene captured by the omnidirectional camera, we use the image calibrated with the principal point for analysis. It is assumed that the ground texture is homogeneous with similar color or pattern. This generally holds for many environments, such as offices and corridors, etc. The edge detection is carried out first, followed by radiating the rays from the image center to reach the edge features. By analyzing the intensity and color distributions of this enclosing region, the image pixels with similar characteristics are clustered. The majority of this cluster corresponds to the ground of the scene, and the image pixels are labeled as the candidates for ground region detection. Since the color features of the ground region are similar, the rays will be excluded if their pixels have a large variation with respect to the overall color distribution. In general, a number of ground detection errors will occur due to the image noise for feature extraction. To deal with this problem, two consecutive images are adopted, and the ground detection results are accumulated to preserve the region with high confidence and remove the isolated rays. Figure 5a shows the detected ground rays overlaid on the original image. In the last stage, the ground detection is derived by filling the adjacent pixels in the regions processed using the image sequence, and the final ground region detection result is illustrated in Figure 5b.

**Figure 6:**
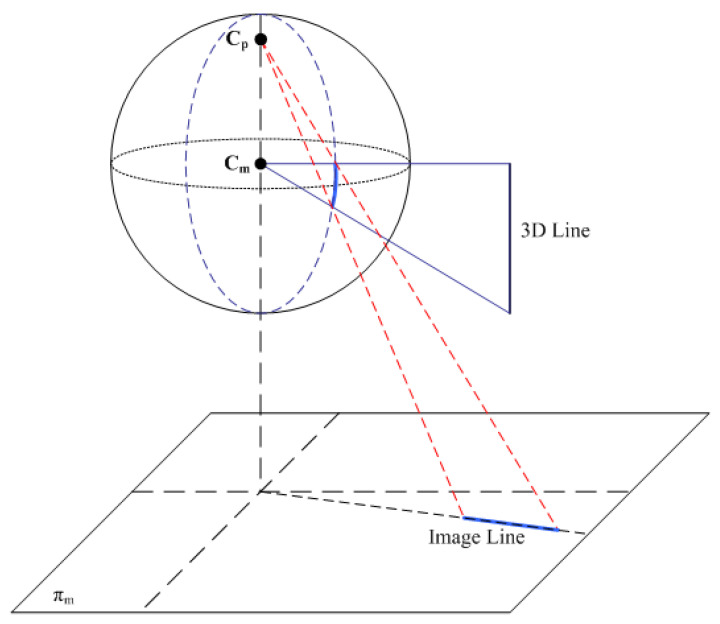
The vertical 3D line in the space is projected to a curve on the unified sphere model via the sphere center at $C_{m}$.

In the applications of mobile robot navigation, the omnidirectional vision system is generally installed perpendicular to the ground, and the hyperbolic mirror faces down to acquire the images of surrounding scenes. One important characteristic of the omnidirectional imaging is the edge features corresponding to the lines perpendicular to the ground in the environment. These feature points form a straight line radiated from the image center regardless of the position of the vertical line in the 3D space. As illustrated in Figure 6, a vertical 3D line in the space is projected to a curve on the unified sphere model via the sphere center at $C_{m}$. This curve is then projected to a line segment on the image plane $\pi_{m}$ via the second center of projection at $C_{p}$. The line segment lies on a ray radiated from the image center, i.e., the projection of $C_{m}$ on the image plane.

**Figure 7:**
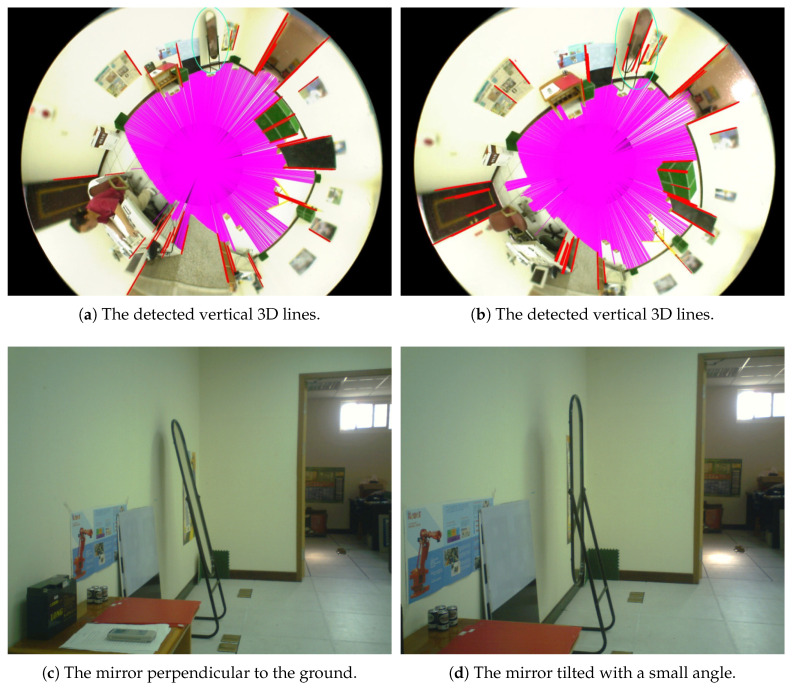
The detection of vertical 3D lines in the space. The red lines in Figure 7a,b indicate the detected vertical 3D lines. The images in which a mirror is placed perpendicular to the ground and tilted with a small angle are shown in Figure 7c,d.

The procedure of our approach to extract the vertical 3D lines in the environment is described as follows. We have the catadioptric vision system mounted on the mobile robot perpendicular to the ground for navigation and image acquisition. This configuration can ensure that the vertical 3D lines are mapped to the radial lines in the omnidirectional image as discussed previously. In [32], Scaramuzza and Siegwart proposed a method to extract the vertical 3D lines in the omnidirectional image. It is relatively expensive computationally in that a descriptor is built exclusively for the vertical line detection. In contrast, Bresenham’s algorithm is adopted in this work to identify the straight line features and improve the line searching speed [33]. The algorithm is modified such that each image pixel is verified once while searching a vertical line along the radial direction in the omnidirectional image. Figure 7 shows the examples of the vertical 3D line detection. The red lines in Figure 7a,b indicate the detected vertical 3D lines in the space. The images in which a mirror is placed perpendicular to the ground and tilted with a small angle are shown in Figure 7c,d. As illustrated by the green circled regions in Figure 7a,b, the proposed method is able to identify the true vertical 3D lines and avoid the features without correct geometric properties.

The proposed technique for the detection of vertical 3D lines in the space is based on the fact that their corresponding line segments in the image can be extended to pass through the image center. This will hold generally, assuming the optical axis of the catadioptric camera is precisely perpendicular to the ground. Since the perfect situation might not always be possible, for example if the ground is not flat, the effect due to a small tilt angle of the vision system is evaluated. It is observed that our algorithm for vertical line detection is able to tolerate up to 3 pixels of the image center offset. Nevertheless, this is not a significant restriction for the applications in indoor environments.

**Figure 8:**
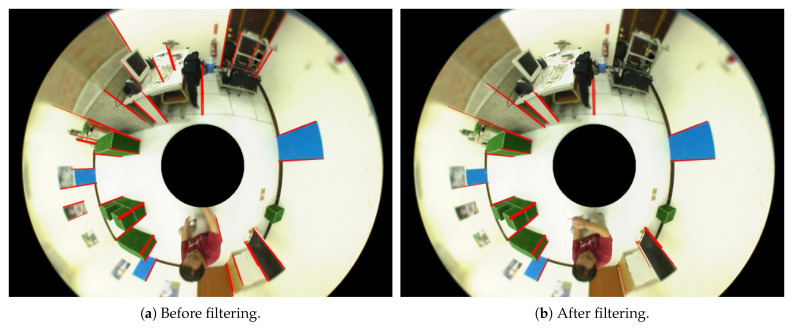
The detection of line segments before and after filtering by the ground plane region.

Given the detection results of the ground plane and vertical 3D lines in the environment, our objective is to find the contact points (or base points) of the vertical structures on the ground. The intersection points between the vertical 3D lines and the ground satisfy the geometric constraints, and will be used to derive the corresponding 3D positions using the calibrated sphere camera model and Equations (5)–(8). However, the endpoints of the line segment detection are not necessarily the true ground points because some vertical structures do not accurately connect to the ground, as illustrated in Figure 8a. To remove the line segments inappropriate for the derivation of base points, those not adjacent to the ground plane region are filtered out. Figure 8b shows the line segments finally used for 3D position estimation. It is clear that some “floating” vertical features are removed from the image for further processing.

### 4.2. Localization and Position Estimation

The mobile robot localization is accomplished by the position estimation using two consecutive frames. Thus, it is required to find the correspondence matching of the base points between two images. In this work, we do not use the SIFT descriptor [34] for robust feature matching due to its high computational cost. By observing the natural or man-made structures in the environment, it is found that some feature points are space invariant during the camera motion. More specifically, the locations and order of the base points in the consecutive images follow some basic pattern rules. Consequently, it is more efficient to track the line segments instead of performing the feature correspondence matching using normalized cross correlation (NCC) [35].

**Figure 9:**
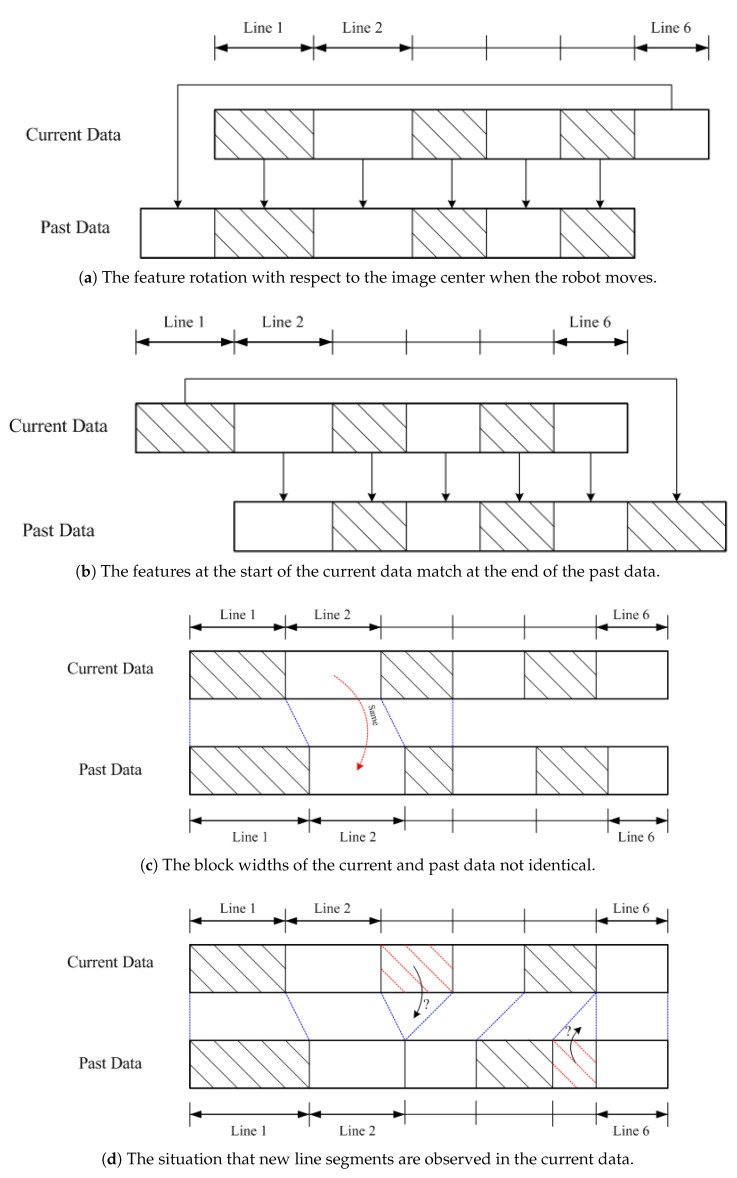
The situations considered to facilitate the line matching between the image frames by tracking. Their orders are always the same since the detected lines are space invariant with respect to the environment.

To facilitate the line matching between the image frames, we consider the situations as shown in Figure 9. In the figure, each block represents a line segment (“Line 1”, “Line 2”, etc.) in the $360^{\circ }$ cyclic order, the width of the block indicates the number of line segments obtained at the same direction, and *Current Data* and *Past Data* correspond to the current and previous image frames, respectively. The first case in Figure 9a shows the common situation of feature rotation with respect to the image center when the robot moves. Due to the construction of the linear and cyclic representation with $360^{\circ }$, it is possible to have the features at the end of the current data match at the start of the past data. Similarly, Figure 9b illustrates the situation where the features at the start of the current data match at the end of the past data. In the real scenes, a vertical 3D line might correspond to multiple detected line segments due to the quality of edge detection algorithms. This makes the block widths of the current and past data not identical, as shown in Figure 9c. To deal with this problem, the line segments are labeled with the orientation, and the matching is carried out using the direction accordingly. Figure 9d shows the situation that new line segments are observed in the current data. In this case, they are not used for matching with the previous image frame but used for the verification of the 3D position of the corresponding base point.

**Figure 10:**
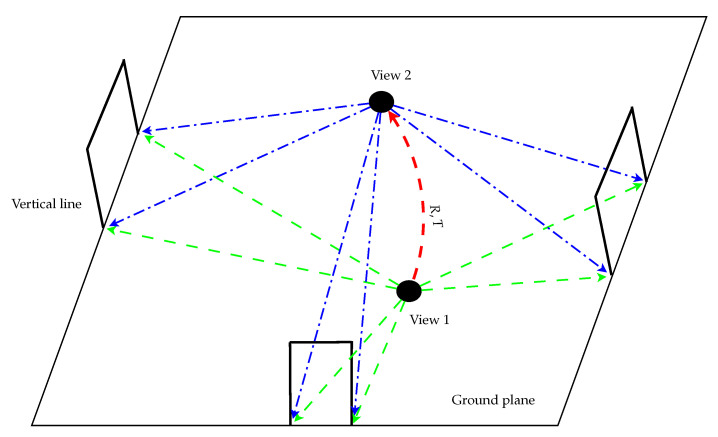
The mobile robot moves from the “View 1” position to the “View 2” with the same set of base points observed. It adopts a catadioptric camera to detect the vertical lines in this structured environment. The base points are used to estimate the 3D information for mobile robot localization.

The conventional stereo matching techniques utilize the disparities obtained from the multi-view cameras to calculate the 3D information. In this paper, only a single omnidirectional camera is used for the acquisition of image sequences. The base points identified in the images are back-projected to the unit vectors on the unified sphere calibrated with the catadioptric camera model. The 3D coordinates of the feature points are then derived from the intersections of the vector extensions and the ground plane equation for robot position estimation. As illustrated in Figure 10, suppose the mobile robot moves from the “View 1” position to the “View 2” position, and the same set of base points observed in these two positions are $\left\{x_{1},x_{2},\cdots ,x_{N}\right\}$ and $\left\{y_{1},y_{2},\cdots ,y_{N}\right\}$, respectively. Then the relative position between “View 1” and “View 2” given by the 3×1 translation vector **T** and the 3×3 rotation matrix R can be derived from

(9) $$y_{i}=Rx_{i}+T$$

where i=1,2,⋯,N. Solving the above equation is equivalent to minimizing

(10) $$\sum_{i=1}^{N}\left|\right|y_{i}-Rx_{i}-T{\left|\right|}^{2}$$

and can be done by singular value decomposition (SVD) [36]. Given multiple robot positions during the navigation and the base point correspondence matching, the localization and motion trajectory can then be computed sequentially.

## 5. Experiments

### 5.1. Experimental Setup

**Figure 11:**
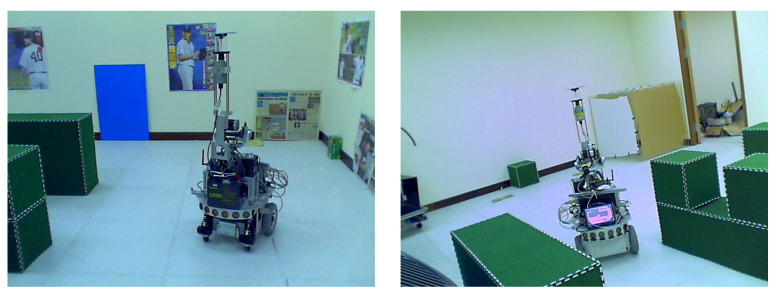
The indoor environment for robot navigation and image acquisition in our experiments.

The proposed techniques have been carried out in the indoor environment for real-world applications. Our mobile platform for the experiment consists of a wheeled robot (U-Bot) equipped with an omnidirectional camera as shown in Figure 1. The catadioptric camera contains a convenient digital camera, SONY DFW-X710, mounted with a hyperboloid mirror with the image resolution of 1024×768. It is able to provide a $360^{\circ }$ field of view in the azimuth plane. An industrial PC with Intel CPU 2.4 GHz, 4 GB RAM is used for onboard real-time processing. Figure 11 illustrates the environment for image acquisition in our experiment. It consists of several obstacles with vertical features which can be used for mobile robot self-localization.

### 5.2. Experimental Results

**Figure 12:**
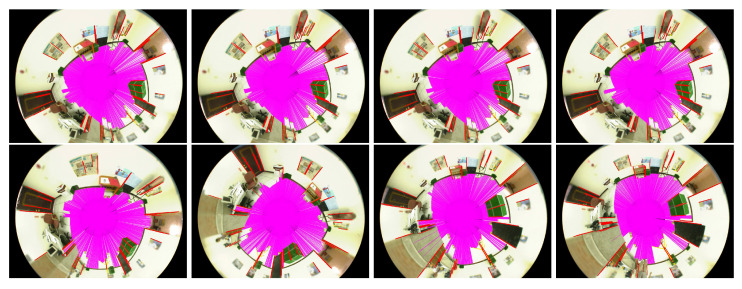
The ground plane segmentation and vertical line identification from an acquired omnidirectional image sequence. It can be seen that the ground region extraction is relatively stable compared to the line segment detection.

In the first experiment, the ground plane segmentation and vertical line identification are carried out in the indoor environment. Figure 12 illustrates the several results derived from the acquired omnidirectional image sequence. It can be seen that the ground region extraction is relatively stable compared to the line segment detection. However, this does not necessarily affect the self-localization results since the ground corners will be identified by the intersection of the ground region and vertical lines. The unstable line segments do not play a key role in the localization computation.

**Figure 13:**
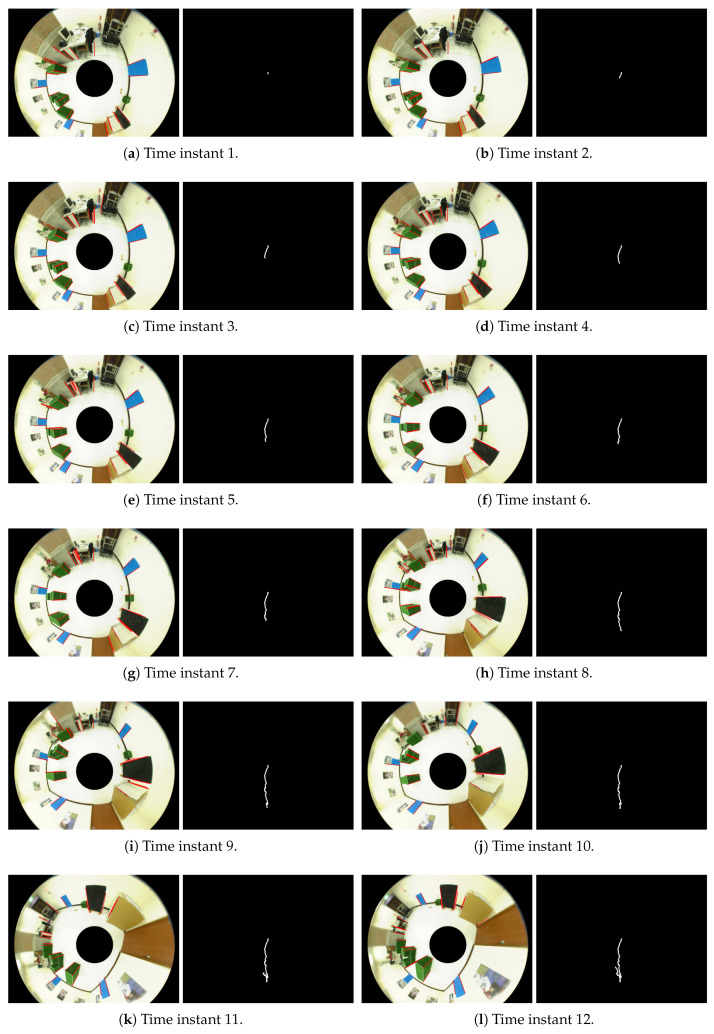
The trajectory of the mobile robot navigation in the indoor environment. It is derived from the proposed self-localization technique using the vertical lines in the space and the corresponding base points on the ground plane.

**Figure 14:**
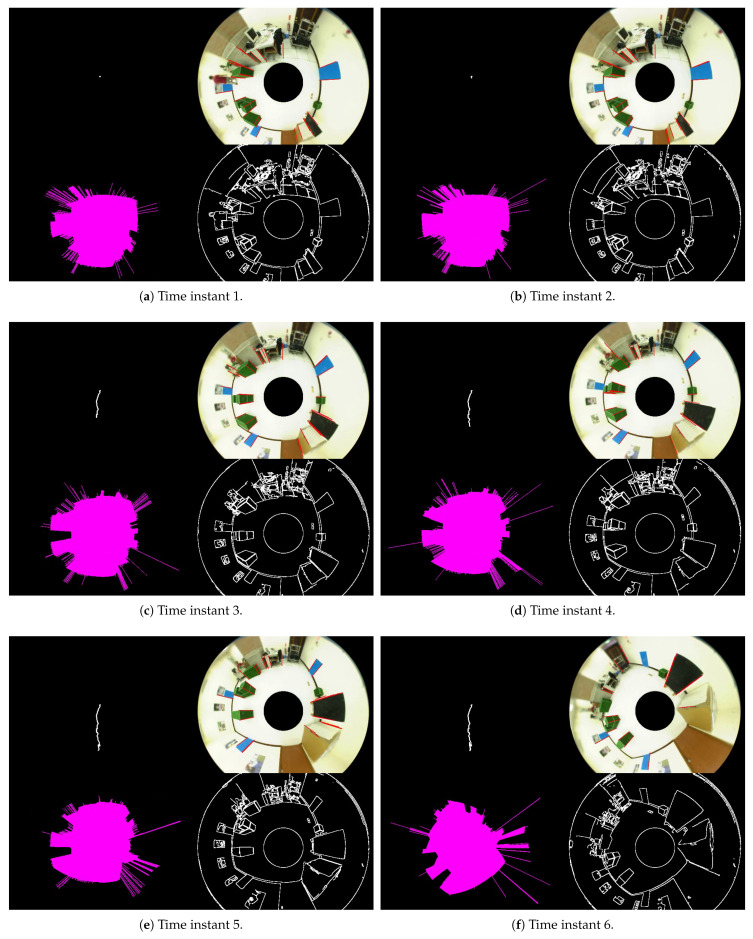
The examination of the localization process. It consists of the vertical line identification, edge detection, ground region segmentation, and trajectory computation for various locations.

The performance of the proposed method is evaluated by measuring the Euclidean distances in the motion trajectory with respect to the mobile robot. For the error analysis, we first verify the similarity of vertical edge features and then compute the errors on the individual lines. As shown in the previous section, the correctness of vertical feature extraction and base point derivation depends on the ground region detection. There might be some errors introduced by the stability of the image acquisition. Table 1 tabulates the position estimates and the estimation errors at several locations. The result indicates that the errors are increased for the base points far away from the mobile robot. This is mainly due to the defocused blur induced by the imaging process through the curved mirror of the catadioptric camera. Figure 13 shows the vertical line extraction and mobile robot self-localization from a sequence of images. There are approximately 20 line segments detected in the omnidirectional images. The results demonstrate that the robot positions can be derived using the geometric constraints and a single catadioptric camera in the experiment. Figure 14 illustrates the examination of the localization process. It consists of the vertical line identification, edge detection, ground region segmentation, and trajectory computation for various locations. In our current implementation in the indoor scene, the experiment is limited to a relatively short distance for navigation. Except for the issues on the available environment, the proposed technique will suffer the drift error if the vertical lines vanish from the viewpoints.

## 6. Conclusions and Future Work

In this paper, we present an approach for the self-localization of mobile robots using a single catadioptric camera. A new technique for vertical line detection and base point identification for the structured environment is proposed. The geometric invariants from the catadioptric image formation are used to perform the base point matching in the image sequence. The derivation of motion trajectory is then carried out based on the computation of rotation and translation between two consecutive robot positions. Our main contributions in this work include the theoretic derivation and framework validation on using a single catadioptric camera for mobile robot localization in structured environments. The experimental results obtained using the real scene images have demonstrated the feasibility of the proposed method. Since the images acquired from the omnidirectional camera contain rich information. In addition to mobile robot self-localization, the future work will focus on the object recognition during navigation. The visual surveillance and human–machine interaction will also be investigated for industrial applications.

## Figures and Tables

**Table 1:** The position estimates and the estimation errors at several locations. The errors are increased for the base points far away from the mobile robot.

Location	Ground Truth	Estimation	Error
1	1000 mm	991.61 mm	−8.39 mm
2	1500 mm	1514.92 mm	14.92 mm
3	2000 mm	2011.18 mm	11.18 mm
4	2500 mm	2440.14 mm	−59.86 mm
5	3000 mm	2844.51 mm	−155.49 mm
6	3500 mm	3340.40 mm	−159.60 mm
7	4000 mm	4240.71 mm	240.71 mm
